# Modulation of Macrophage Activities in Proliferation, Lysosome, and Phagosome by the Nonspecific Immunostimulator, Mica

**DOI:** 10.1371/journal.pone.0117838

**Published:** 2015-02-10

**Authors:** Myunghwan Jung, Min-Kyoung Shin, Yeon-Kwon Jung, Han Sang Yoo

**Affiliations:** 1 Department of Infectious Diseases, College of Veterinary Medicine, Seoul National University, Seoul, Republic of Korea; 2 Seobong BioBesstech Co., Ltd., Yeoksam-dong, Kangnam-gu, Seoul, Republic of Korea; 3 Institute of Green Bio Science and Technology, Seoul National University, Pyeongchang, Republic of Korea; Cornell University, UNITED STATES

## Abstract

It was reported that the aluminosilicate material mica activated macrophages and showed its immunostimulating effects. However, the mechanisms by which it exerts these effects are unclear. To address this, we evaluated the effects of mica fine particles (MFP, 804.1 ± 0.02 nm) on the murine macrophage cell line, RAW 264.7. Specifically, RAW 264.7 cells were treated with 100 and 500 μg/mL MFP and their proliferative response was determined using the 3-(4,5-dimethylthiazol-2-yl)-2,5-diphenyltetrazolium bromide (MTT) assay. Changes in global gene expression upon MFP treatment for 12 and 48 h were also determined using microarrays. Following the MFP treatment, RAW 264.7 cells showed a low level of proliferation compared to nontreated cells (*p* < 0.01). There was a change in an expression level of 1,128 genes after 48 h treatment. Specifically, genes associated with the cell cycle, DNA replication, and pyrimidine and purine metabolisms, were down-regulated in cells treated with MFP, which resulted in reduction of cell proliferation. MFP treatment also up-regulated genes associated with lysosome and phagosome function, which are both required for macrophage activities. We speculate that activation of macrophages by mica is in part derived from up-regulation of these pathways.

## Introduction

Mica, which is well known for its immunostimulating effects, is a common aluminosilicate mineral containing potassium, magnesium, iron, aluminum, and silicates. The immunostimulating effects of mica have been proven through many previous studies [1–5]. Mica has been used as feed supplement to improve immune activities [3–5] and increase an absorption rate of high-protein nutrients [1, 2]. As described in the above study results, most studies using mica have been concentrated on the proof of the immune enhancement effects but few studies have been done on the mechanism of how the immunity enhancement effect is induced. It was reported that mica activated macrophages and the immunity enhancement effect was induced by this activation of the macrophages [6]. In addition, a number of studies have disclosed that mica acted as a superantigen, leads to T-cell activation and promotes phagocytosis of macrophages through high affinity binding to major histocompatibility complex (MHC) class II molecules. This in turn leads to immune system activation *via* the induction of proinflammatory cytokines such as interleukin IL-1α, IL-6, and TNF-α [7–9]. Nonetheless, the precise mechanism by which mica modulates macrophage function and induces their activation remains to be determined.

Macrophages are critical effectors of the immune response, and carry out the removal of ‘non-self’ material *via* phagocytosis [10]. They are considered one of the main entry routes of particles into the body, and thereby play an important role in determining the biopersistence of foreign particles and in the associated inflammatory responses triggered by their phagocytic activities [11, 12]. They have been also known to participate in innate immunity and in the first line of immune defense [13]. Previous studies suggested that macrophages could interact with T-cells using cytokines secretion and receptors thereby modulating the immune responses [10, 14].

In this study, to investigate the mechanisms associated with mica-dependent activation of macrophages, we manufactured mica fine particles (MFP) and used them to treat the murine macrophage cell line, RAW 264.7. Following the MFP treatment, global cell responses were investigated using a microarray approach. Through this genome-wide approach, we were able to infer which cellular signaling pathways are engaged following MFP treatment.

## Materials and Methods

### MFP preparation

The MFP were produced by Seobong Biobestech (Seoul, Korea), containing silicon dioxide (61.90%), aluminum dioxide (23.19%), iron oxide (3.97%), sodium oxide (3.36%), calcium oxide (< 2%), magnesium oxide (< 2%), titanium oxide (< 2%), and 36 ppm germanium.

### Morphology and size of MFP

The morphology of the MFP was observed using a field emission scanning electron microscope (FE-SEM; JSM-6700F, JEOL, Tokyo, Japan) following platinum-coating using Cressington 108 (Cressington, Watford, UK). The particle size of MFP was measured using the DLS-7000 spectrophotometer (Otsuka Electronics Ltd., Osaka, Japan).

### Cell preparation and evaluation of effect of MFP on cell proliferation

The murine leukemic monocyte macrophage line, RAW 264.7, was obtained from Korea Cell Line Bank (KCLB No. 40071; Seoul, Korea) and grown at 37°C in a 5% CO_2_ atmosphere in Roswell Park Memorial Institute medium (RPMI; Gibco, Carlsbad, CA, USA) 1640 containing 10% FBS (Gibco). The cells were then seeded at a concentration of 8.0 × 10^4^ cells/cm^2^ in 12-well plates with 1 mL of media containing 2% FBS. Following 8 h culture, cells were incubated with 100 or 500 μg/mL of MFP for 0, 12, 24, or 48 h. After treatment with MFP, the cells were then incubated in FBS-free media with 0.5 mg/mL of (3-(4,5-dimethylthiazol-2-yl)-2,5-diphenyltetrazolium bromide (MTT; Life Technologies, Carlsbad, CA, USA) for 4 h. Formed crystals were dissolved in 1 mL of dimethyl sulfoxide (DMSO; Sigma, USA) and 100-μL aliquots were transferred to 96-well plates. The plates were read on an Emax Precision Microplate Reader (Molecular Devices, Sunnyvale, CA, USA), using a test wavelength of 590 nm and a reference wavelength of 620 nm. Cell numbers were calculated based on the standard curve generated from serially diluted cells. The effect of MFP on cell proliferation was expressed relative to the cell number at 0 h.

### RNA preparation

RAW 264.7 cells cultured in RPMI 1640 containing 10% FBS were seed at a concentration of 8.0 × 10^4^ cells/cm^2^ in T75 flasks with 20 mL of media containing 2% FBS. Following an 8-h culture, cells were incubated with 100 μg/mL of MFP and collected at 0, 12, and 48 h post-stimulation. RNA was extracted using the RNeasy mini kit (Qiagen, Venlo, Limburg, Netherlands) as described by the manufacturer. All RNA samples were quantified, aliquoted, and stored at -80°C until use. Purity and integrity of RNA samples was determined using denaturing gel electrophoresis, OD 260/280 ratio, and analysis on an Agilent 2100 Bioanalyzer (Agilent Technologies, Palo Alto, CA, USA).

### Microarray hybridization

RNA amplification, labeling, array hybridization, and scanning were carried out by Macrogen Inc. (Seoul, Korea). Amplification and purification of total RNA (550 ng) was performed using the Ambion Illumina RNA amplification kit (Ambion Inc., USA) per the manufacturer’s recommendations in order to obtain biotinylated cRNA. Following quantification of the cRNA using the ND-1000 Spectrophotometer (Thermo Scientific, Wilmington, DE, USA), 750 ng of labeled cRNA samples were hybridized to each Illumina expression beadchip (Mouse WG-6 v2.0; Illumina Inc., San Diego, CA, USA) for 16–18 h at 58°C, which covers more than 45,200 transcripts, according to the manufacturer’s instructions. After the bead array manual, detection of array signals was performed using Amersham Fluorolink Streptavidin-Cy3 (GE Healthcare Bio-Sciences, Little Chalfont, UK). Arrays were scanned with an Illumina bead array Reader confocal scanner (Illumina Inc.) as described by the manufacturer.

### Raw data preparation and statistical analysis

The quality of hybridization and overall chip performance were monitored by visual inspection of an internal quality control check and the raw scanned data. Raw data were extracted using the Illumina GenomeStudio v2009.2 (Gene Expression Modulev1.5.4, Illumina Inc.) and selected based on a *p*-value < 0.05 with no fail-count (sample count of detection *p*-value ≥ 0.05). These selected gene signal values were transformed by logarithm and normalized by the quantile method. The comparative analysis between the test and control samples was performed using fold change. Genes showing more than 2-fold increase or decrease were considered to be significantly altered. All data and visualization of differentially expressed genes were carried out using R2.4.1 (www.r-project.org).

### Microarray data analysis

Gene set enrichment analysis for genes showing significant altered expression was performed using Protein Analysis Through Evolutionary Relationships (PANTHER) (http://www.pantherdb.org). Differentially expressed genes were categorized by biological process and molecular function using the PANTHER classification database by means of Fisher’s exact test to detect coordinated changes in pre-specified sets of related genes. Clustering analyses of differently expressed genes were investigated using MultiExperiment Viewer version 4.9.0 software (MeV; Institute for Genomic Research, Boston, MA, USA). Based on these altered genes, unsupervised hierarchical clustering was conducted to determine the relation according to MFP treatment period. In addition, the patterns of changes in gene expression were also analyzed through quality threshold clustering (QTC) method.

The changes in cell process are derived from pathways formed by the interaction of genes [15, 16]. Therefore, Kyoto Encyclopedia of Genes and Genomes (KEGG) pathway mapping of genes involved each cluster was also carried out in order to analysis systemic information representing functional aspects of each of patterns (http://www.genome.jp/kegg/tool/map_pathway2.html). For a pathway mapping term to be considered significantly, pathway represented by fewer than 10 genes were filtered out, thereby identifying the most robustly affected pathways [11]. Dataset of microarray results has been deposited in Gene Expression Omnibus (GEO, http://www.ncbi.nlm.nih.gov/geo/query/acc.cgi?acc=GSE63827) and accessible through GEO series accession number GSE63827.

### Verification of microarray results

To verify the microarray results, 3 genes that were identified as being up-regulated and associated with lysosome and/or phagosome pathways and 4 genes that showed down-regulation and involved in cell cycle, pyrimidine metabolism, and/or purine metabolism (Table 1) were randomly selected and subjected to quantitative real time RT-PCR (qRT-PCR). Total RNA (1 μg), the remainder of microarray analysis, was submitted for synthesis of cDNA using QuantiTect Reverse Transcription kit (Qiagen) according to the manufacturer’s protocol. The qRT-PCR reaction was carried out with 1 μL of cDNA using the Rotor-Gene SYBR Green PCR kit (Qiagen) and Rotor-Gene Q real time PCR cycler (Qiagen). The cDNA was amplified under following conditions: 45 cycles at 95°C for 15 s followed by 45 s at 60°C. The fluorescence was detected during the extension phase and the expression level was compared to a non-stimulated RAW 264.7 cell control by the 2^-∆∆C^_T_ method using the house-keeping gene, glyceraldehyde-3-phosphate dehydrogenase (GAPDH), as a reference [17].

**Table 1:** Primer used for qRT-PCR.

Genes		Primers (5'-3')	Pathway	Accession no.
Pla2g1	F	CAGTTTCCCGATGGTGTGGA	Lysosome	NM_133792.2
	R	CCGTTTTCATTTGGGGCTCG		
Lamp2	F	GTTCCTAGGAGCCGTTCAGTC	Lysosome and phagosome	NM_001017959.1
	R	TCATCCCCACAACTGCTTCC		
Arsb	F	GTGCGCCGATTGAGTCTTTG	Lysosome	NM_009712.3
	R	AACAGTGGTTTCTCCGGTGG		
Nme1	F	GACCGCCCCTTCTTTACTGG	Pyrimidine and purine metabolisms	NM_008704.2
	R	CCTCCCAGACCATAGCAACC		
Rrm1	F	ACGAAGCACCCTGACTATGC	Pyrimidine and purine metabolisms	NM_009103.2
	R	TGGCAGAATTCAGGCGATCC		
Ccne1	F	TTCGGGTCTGAGTTCCAAGC	Cell cycle	NM_007633.2
	R	TGCAAAAACACGGCCACATT		
Espl1	F	CAAGCCGCGACTTTTGCC	Cell cycle	NM_001014976.1
	R	GCAAGCCCTCAGGATGGTAT		

### Statistics

Data are presented as mean ± standard deviation (SD). Statistical analyses were performed by using the Student’s *t*-test or repeated measures of ANOVA using SPSS version 19.0 software (SPSS, USA). The statistical significance of differences was set at value of *p* < 0.05.

## Results

### Morphology and size of MFP

**Fig 1:**
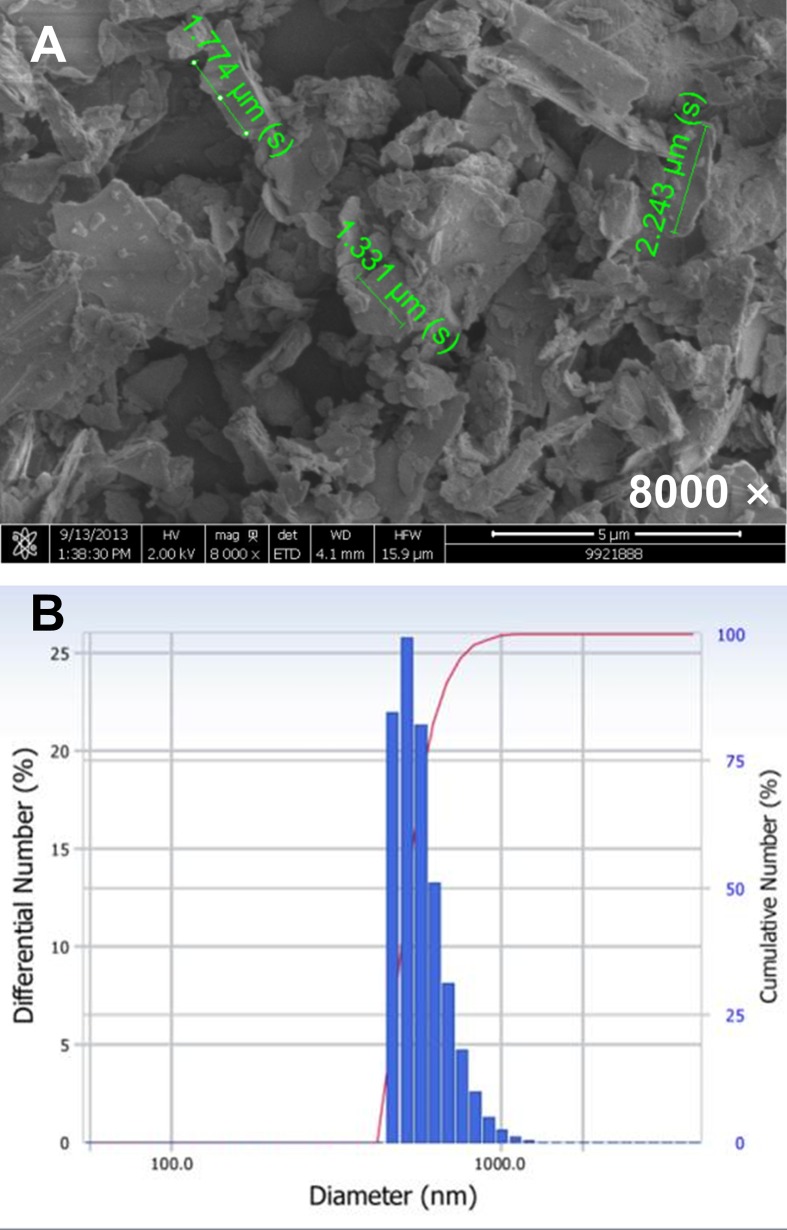
SEM microphotographs (A) and the diameter distribution of mica fine particles (B).

SEM micrographs revealed that MFP were irregular polygons with a rough surface. The average MFP particle size was 804.1 ± 0.02 nm, which places these substances in the ‘fine particles’ class as described by the United States Environmental Protection Agency (Fig. 1).

### Effect of MFP on cell proliferation

**Fig 2:**
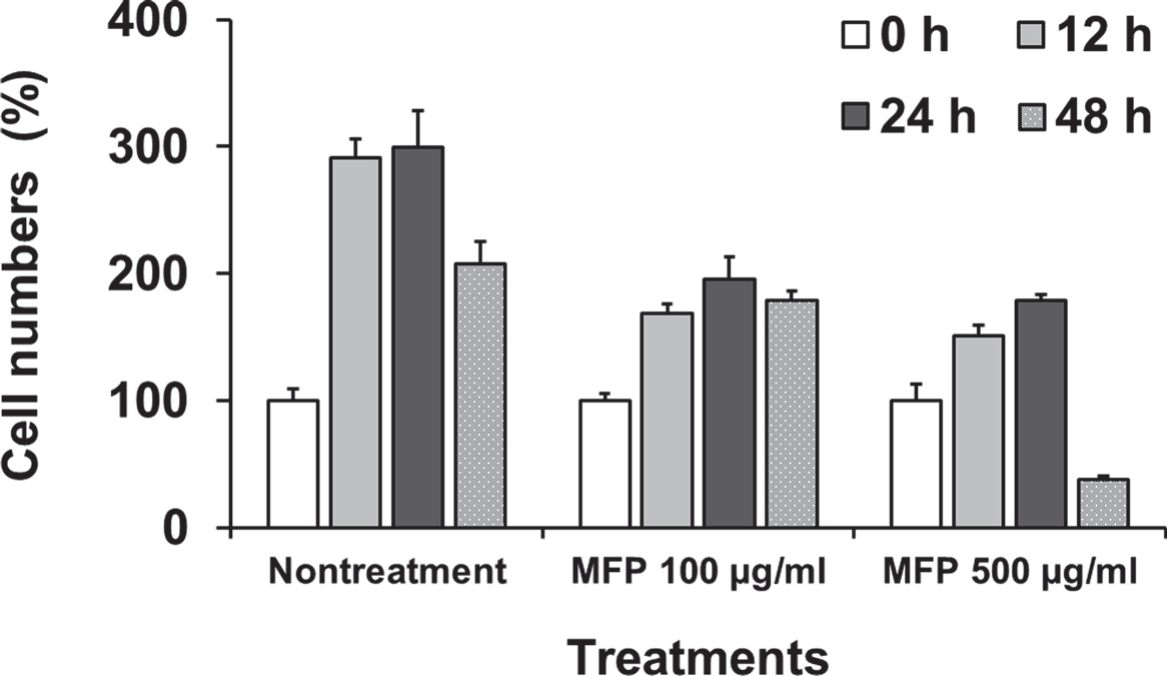
Proliferation of RAW 264.7 cells after treatment with 100 and 500 μg/mL of MFP. Cell proliferation was measured using the MTT assay.

MFP-treated cells showed proliferative responses until 24 h (Fig. 2). All MFP-treated cells showed significant increases in cell numbers except treatment with 500 μg/mL for 48 h which showed significant decrease (*p* < 0.01). Nontreated cells demonstrated same proliferation pattern as MFP 100 μg/mL treatment, showing high level of proliferative responses compared to the MFP treatment (*p* < 0.01). An appropriate concentration was defined as 100 μg/mL at which a significant decrease of cell population was not observed for 48 h. In addition, we defined early and late treatment time points as 12 h and 48 h, respectively.

### Microarray analysis of differentially expressed genes after stimulation with MFP

**Fig 3:**
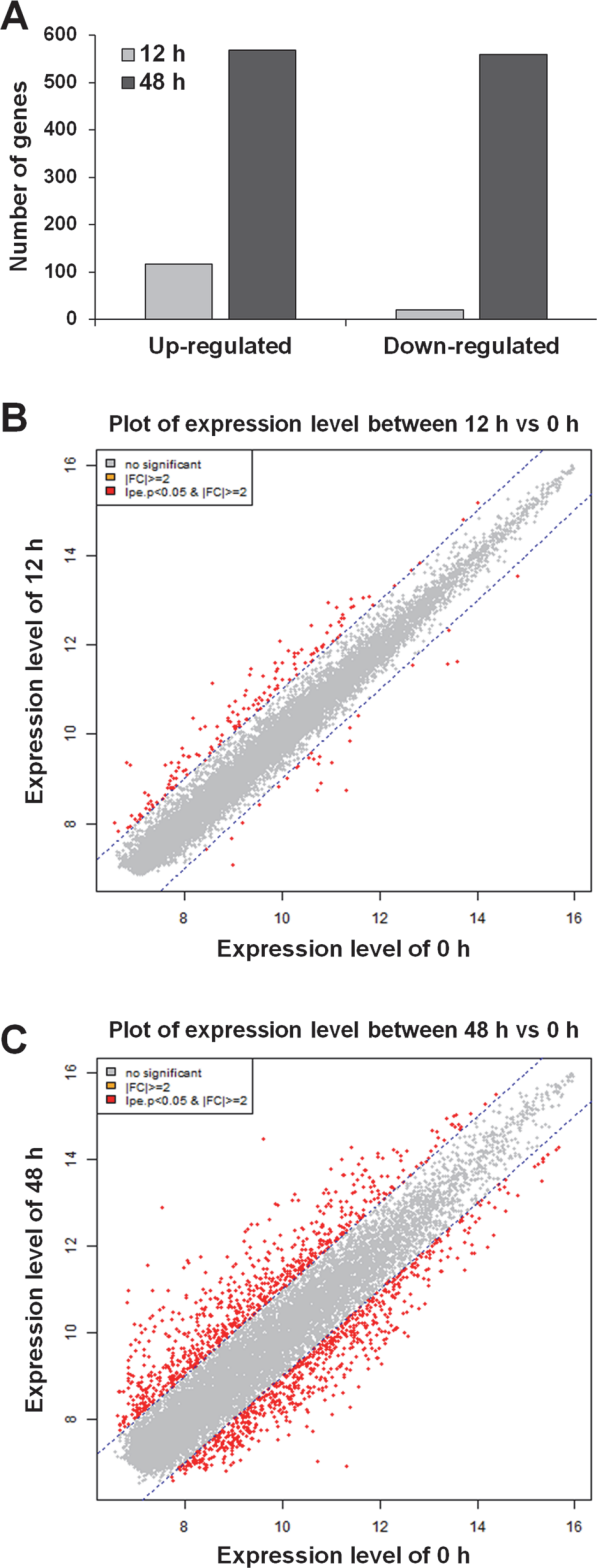
Different gene expression after treatment with 100 μg/mL of MFP. (A) Count of up-and down-regulated genes in RAW 264.7 cells treated with MFP 100 μg/mL compared to nontreated. (0 h; *p* < 0.05, |fold change | ≥ 2). (B) and (C) Plots of the expression level between nontreated cells versus those treated with 100 μg/mL MFP for 12 or 48 h. Red dots present an expression level change of ≥ 2 or ≤ -2-fold for both up- and down-regulated genes. Expression levels were calculated by base 2 logarithm of normalized hybridization signals from each sample.

Following MFP treatment for 12 h and 48 h, we observed greater than 2-fold changes in expression of 1,165 of 30,854 genes examined. Among the 1,165 genes, 116 and 569 genes were up-regulated, and 20 and 559 genes were down-regulated at 12 h and 48 h, respectively (Fig. 3A). Fig. 3B and 3C shows the median of normalized hybridization signals of the genes with altered transcription between the MFP 100 μg/mL treated cells for 12 h or 48 h and for 0 h. Red dots indicate an expression level change of ≥ 2 or ≤ 2-fold for both up- and down-regulated genes with *p* < 0.05. As shown in the graph, a larger number of red dots, indicating more than 2-fold changes in expression, were observed in the MFP-treated cells at 48 h compared to treatment for 12 h.

### Gene set enrichment analysis

**Fig 4:**
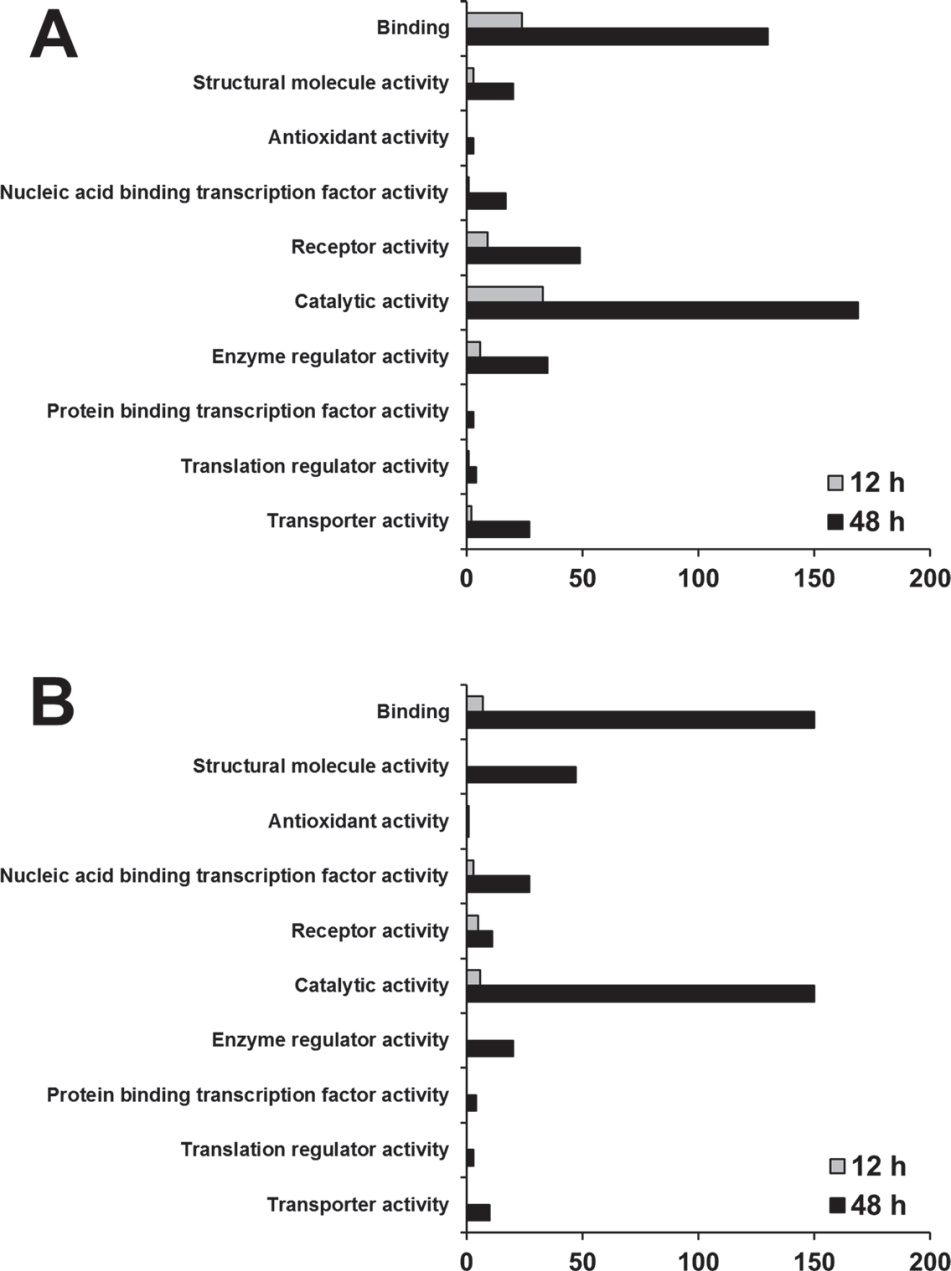
Categorization by molecular function of genes showing significant regulation. Up-regulated transcripts (A) and down-regulated transcripts (B) in RAW 264.7 macrophage at 12 h and 48 h post treatment with 100 μg/mL MFP. (*p* < 0.05, Fisher’s exact test)

**Fig 5:**
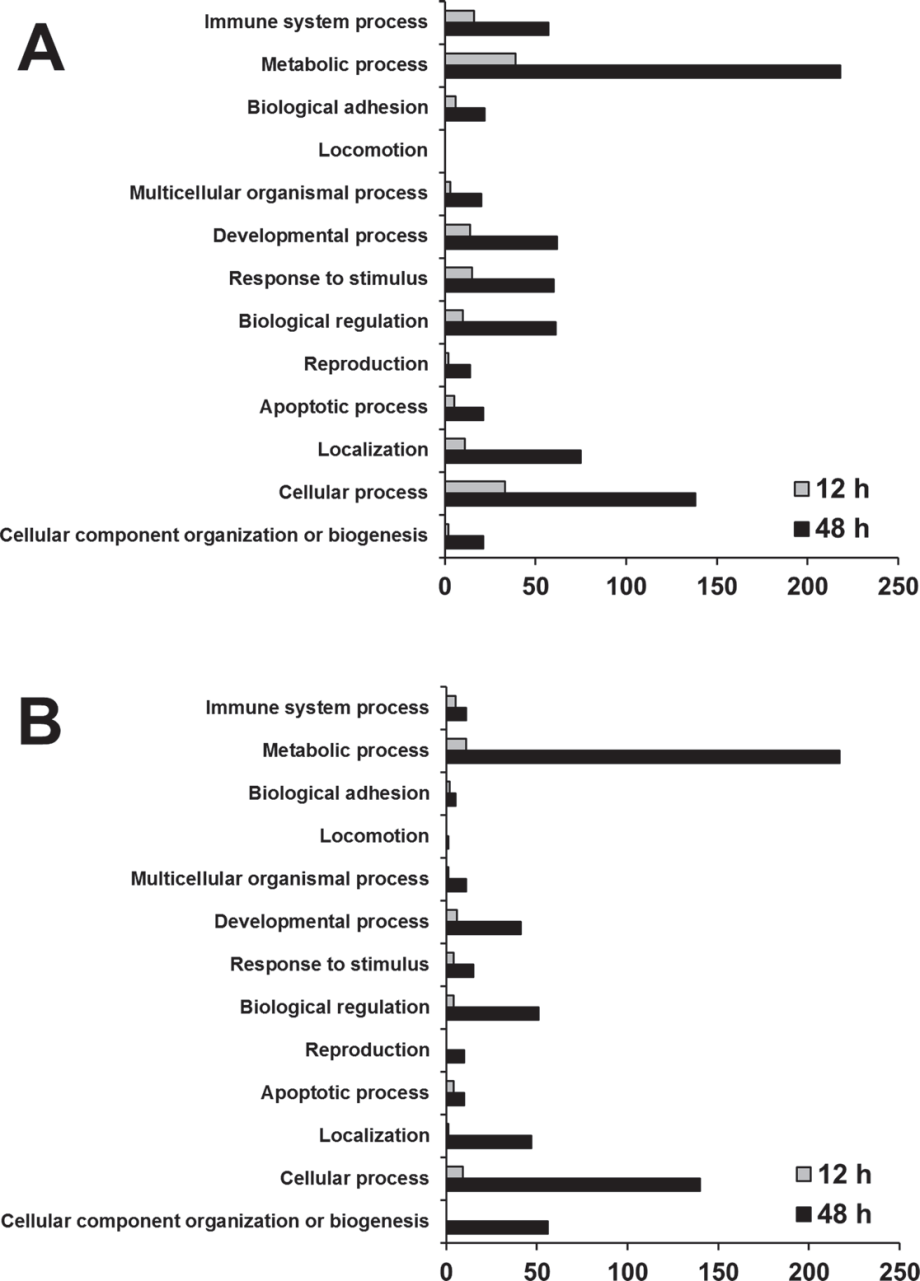
Categorization by biological process of genes showing significant regulation. Up-regulated transcripts (A) and down-regulated transcripts (B) in RAW 264.7 macrophage at 12 h and 48 h post treatment with 100 μg/mL MFP. (*p* < 0.05, Fisher’s exact test)

Each of the up- and down-regulated genes were categorized by using gene set enrichment analysis using the PANTHER classification database to detect coordinated changes in pre-specified sets of related genes. This revealed that 10 molecular function categories (Fig. 4) and 13 biological process categories (Fig. 5) were associated with transcriptional changes following treatment with MFP 100 μg/mL. Most of the differentially up- and down-regulated genes were involved in two molecular function categories (binding and catalytic activity) and two biological process categories (metabolic and cellular processes).

### Clustering Analyses

**Fig 6:**
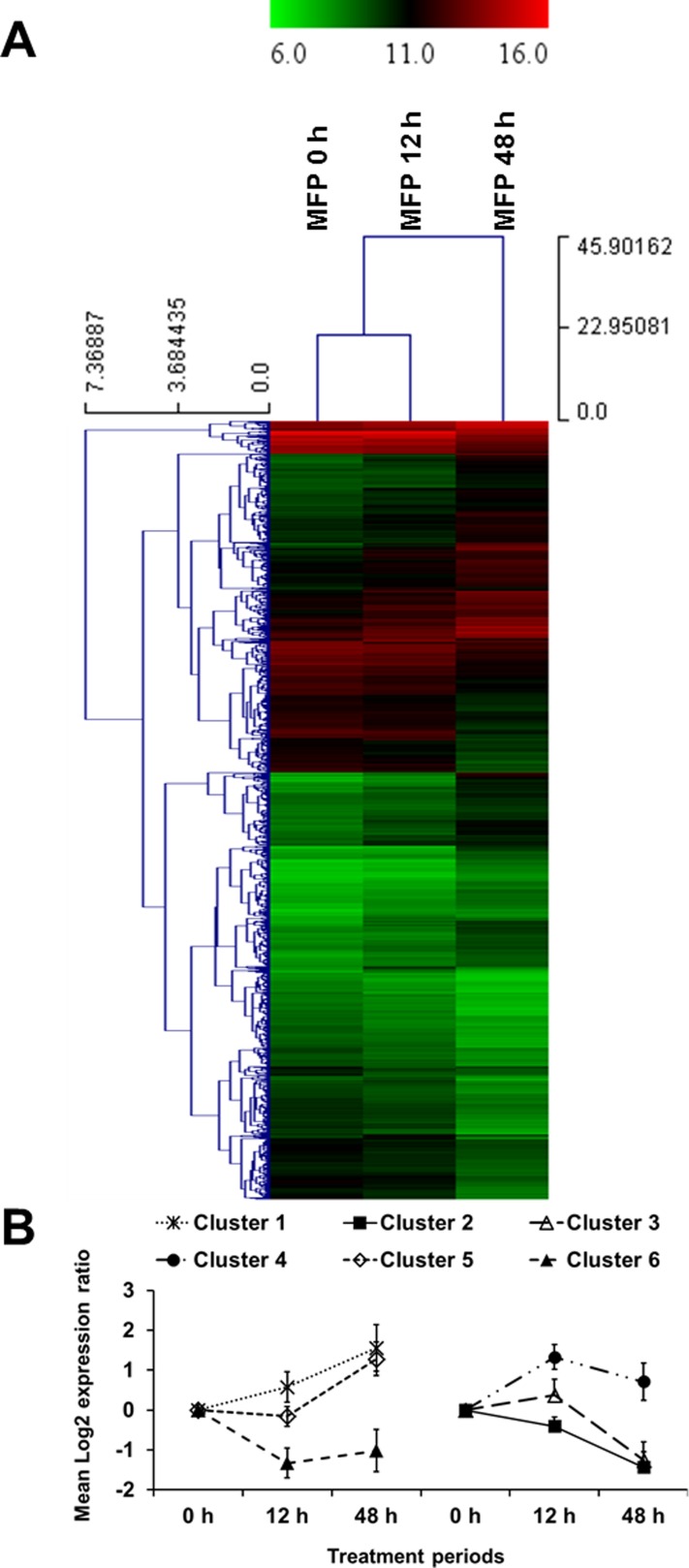
Unsupervised hierarchical clustering (A) and expression pattern profiles according to quality threshold clustering (B). The clustering results were determined using differentially expressed genes after treatment with 100 μg/mL of MFP. (*p* < 0.05, |fold change | ≥ 2)

The 1,165 genes that were differentially expressed were subjected to unsupervised hierarchical clustering. As shown Fig. 6A, experimental groups could be divided into two groups by 0 and 12 h versus 48 h based on gene expression. QTC analysis revealed that gene expression patterns according to MFP treatment were classified under six clusters (Fig. 6B). Cluster 1, 2, 3, 4, 5, and 6 contained 524, 504, 57, 36, 34, and 10 genes, respectively. Cluster 1 and 2 presented the genes showing continuous up- and down-regulation, respectively. The genes involved cluster 3 and 5 showed no noticeable expression changes at 12 h, however, down- and up-regulation were observed at 48 h in two clusters, respectively. The genes showing up-regulation at 12 h and then down-regulation at 48 h were classified as cluster 4. Cluster 6 demonstrated the genes that showed no noticeable expression change since down-regulation at 12 h. The clustering result of all genes showing altered expression is provided in S1 Table.

### Analyses of affected pathways

**Fig 7:**
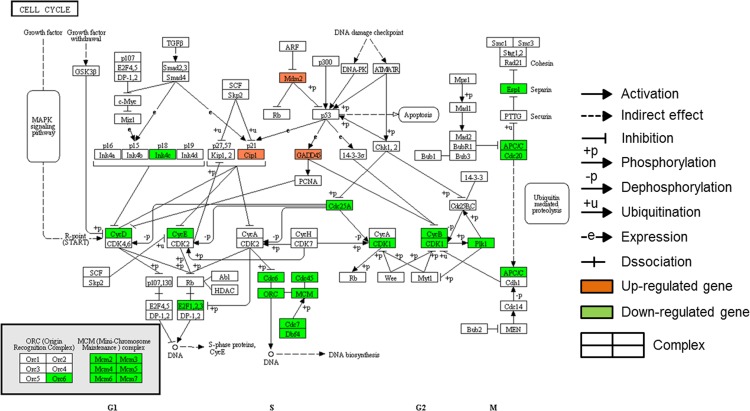
Genes showing altered expression in the cell cycle pathway after treatment of MFP 100 μg/mL. Activation of p53 enhances expression of Gadd45 and Cip1 (*Cdkn1a*). Up-regulation of Gadd45 and Cip1 inhibit CycD (*Ccnd3*). CycE (*Ccne1* and *2*) and CycB (*Ccnd3*) are also down-regulated by Cip1 and Gadd45, respectively. Reduced expression of CDK1 (*Cdc2a*) leads to down-regulation of Plk1, which has critical function during mitosis. In addition, down-regulation of Cdc6, ORC (*Orc6l*), Cdc45 (*Cdc45l*), MCM, and EfF2 result in reduction of DNA synthesis. Reduced expression of APC/C (*Anapc5*) and Cdc20 down-regulates expression of Espl (*Espl1*), thereby affecting on chromosome segregation. This pathway map was conducted using KEGG mapper.

**Fig 8:**
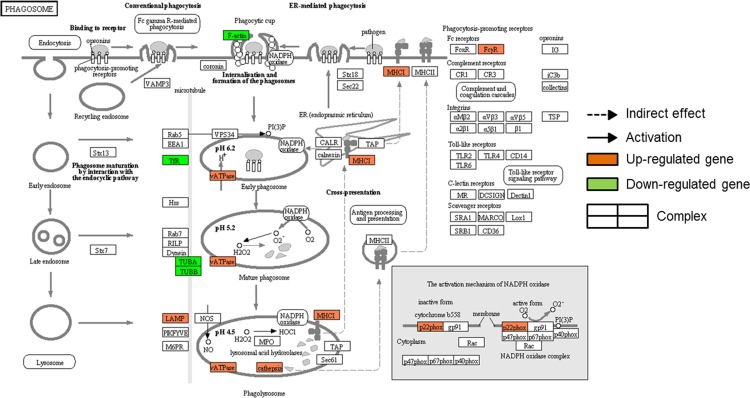
The genes showing altered expression in the phagosome pathway after treatment of MFP 100 μg/mL. The MFP treatment down-regulated genes associated with microtubule activity such as F-actin (*Actb*), TfR (*Tfrc*), TUBA (*Tuba1a* and *1b*), and TUBB (*Tubb2c*, *5*, and *6*). Activation of NADPH oxidase by up-regulation of p22phox (*Cyba*) enhances catabolic activities in phagosome. vATPase (*Atp6v0a1*, *Atp6v1a*, *Atp6v1d*, and *Atp6v1g1*), which was up-regulated by MFP treatment, plays an critical role in receptor-mediated endocytosis by providing the acidic endosomal environment in phagosome. As proteases, cathepsin (*Ctsl* and *Ctss*) were also up-regulated by MFP treatment. MHC І (*H2-D1*,-*K1*,-*Q6*,-*Q7*,-*Q8*, and-*T23*) and FcγR (*Fcgr1*, *2b*, *3*, and *4*), which play role in phagosome activation as antigen presenting molecules and phagocytosis-promoting receptors, were also up-regulated by MFP treatment. This pathway map was conducted using KEGG mapper.

In order to elucidate whole chains of events caused by differently expressed genes involved each cluster of QTC, KEGG pathway mapping was conducted with significant regulated genes in each cluster. Among pathways constructed using the differentially expressed genes classified as cluster 3, 4, 5, and 6, there was no pathway represented by more than 10 genes. In case of differentially regulated genes involved cluster 1 and 2, total 114 genes were listed on 9 pathways (S2 Table). Pathways of cell cycle, lysosome, phagosome, pyrimidine metabolism, purine metabolism, cell adhesion molecules, DNA replication, endocytosis, and antigen processing and presentation were represented by 26, 24, 21, 19, 16, 14, 16, 11, and 10 genes, respectively. In addition, lysosome, phagosome, cell adhesion molecules, endocytosis, and antigen processing and presentation pathways were mainly associated with cluster 1 whereas cell cycle, purine metabolism, pyrimidine metabolism, and DNA replication pathways were represented by mainly genes of cluster 2. Figs. 7 and 8 conducted using KEGG mapper show the pathways of cell cycle and phagosome, respectively. The genes of *Gadd45*, *Cip1* (*Cdkn1a*), and *Mdm2* involved in cell cycle pathway showed gene expression pattern of cluster 1 (Fig. 7). In addition, down-regulation in gene expression associated with microtubule activity was observed in Fig. 8.

### Validation of microarray data

**Fig 9:**
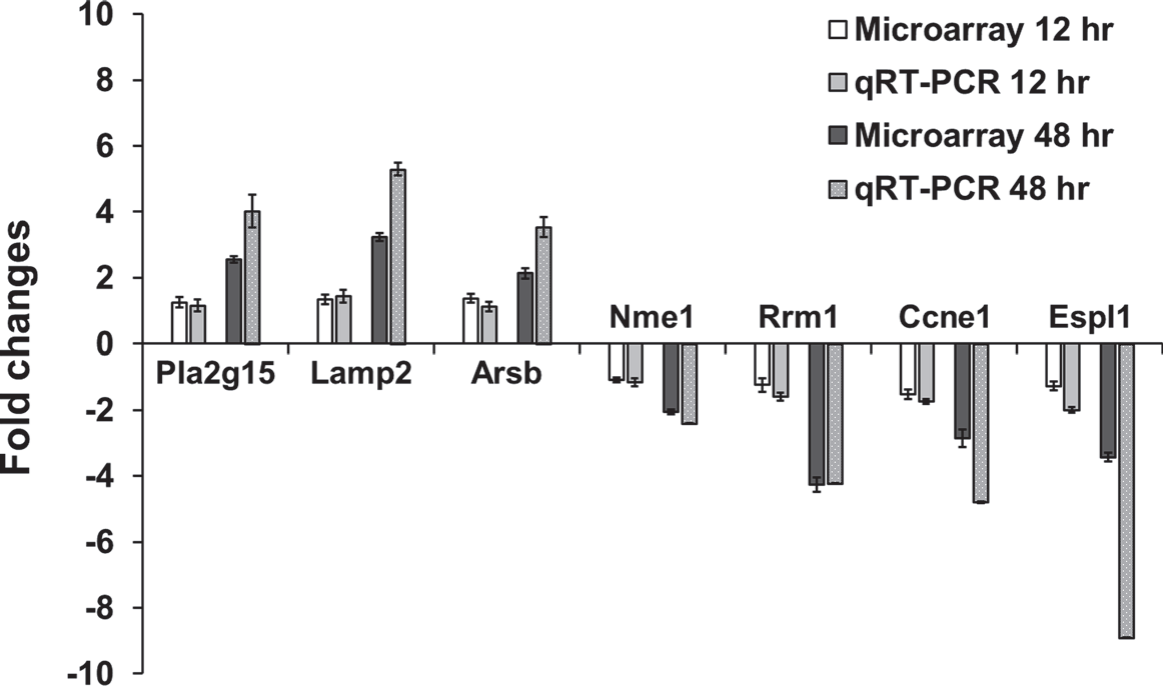
Validation of microarray data *via* quantitative RT-PCR. The relative expression level was normalized to the level of GAPDH expression using the 2^-ΔΔCT^ method.

To verify the microarray results, qRT-PCR was also conducted using the same experimental RNA samples with 7 selected genes that showed altered expression and involved lysosome, phagosome, cell cycle, pyrimidine metabolism, and/or purine metabolism functions. As shown Fig. 9, all genes tested by qRT-PCR showed same direction in expression levels as the microarray results.

## Discussion

The immune enhancement effects of mica are clear based on the results of several previous studies [3–7, 9]. Aluminosilicate, one of the major ingredients of mica, is known to play an important role in stimulating an immune response *via* inducing the activation of macrophages [4, 6, 7, 9]. Nonetheless, few studies have addressed the mechanism of macrophage activation by mica. Our current analysis of the macrophage response to mica is, to our knowledge, one of the first to infer macrophage signaling networks that are modulated by mica treatment.

As shown in Fig. 3, the number of genes that showed changes in gene expression level at 48 h increased more than those of 12 h. In addition, unsupervised hierarchical clustering results showed that gene expression pattern of 0- and 12-h treated cells formed high level of similarity compared to 48 h. On the other hand, gene expression at 48 h was different from those of 0- and 12-h treated cells. Together these data reveal that a 12-h MFP treatment has no noticeable effect on gene expression, whereas a 48-h treatment elicits significant changes. The QTC analysis showed that differently expressed genes by MFP treatment could be classified into 6 clusters. As the results, most of up- and down-regulated genes were involved in cluster 1 and 2; therefore, it could be thought that MFP-treated cells were mainly affected by the genes of these clusters. The KEGG mapping analysis also supported this suggestion. All of the identified pathways, which are represented by more than 10 genes, were mostly conducted with the genes involved cluster 1 and 2. Based on the results, it was determined that the pathways of cell cycle, lysosome, phagosome, pyrimidine metabolism, purine metabolism, cell adhesion molecules, DNA replication, endocytosis, and antigen processing and presentation were significantly affected by MFP treatment.

Among the identified pathways, the cell cycle pathway, which contained the largest number of genes showing different expression, is involved in cell division and proliferation (Fig. 7). The key regulatory enzymes in the cell cycle pathway are cell division cycle kinases (*Cdc*s) and cyclin-dependent kinases (*CDK*s) [18–20]. Genes related to the cell cycle pathway, such as *Cdkn2c*, were generally down-regulated after MFP treatment, and exhibited an expression pattern that placed them in cluster 2. On the other hand, *Cdkn1a*, *Gadd45*, and *Mdm2*, which are cell cycle-related genes that were all up-regulated, were classified into cluster 1. This result was thought that up-regulation of *Cdkn1a*, *Gadd45*, and *Mdm2* was associated with the p53 pathway. It was known that the tumor suppressor p53 plays an important role in cellular stress response pathway [21–23]. p53 activity is kept low under non-stressed conditions by its predominant negative regulator, *Mdm2* [21]. However, under certain circumstances such as cell stress, drug treatment, or hypoxia, p53 protein is accumulated and is activated in order to prevent uncontrolled growth. *Cdkn1a* and *Gadd45*, as target genes of p53 pathway, were up-regulated according to activation of the p53 pathway, thereby down-regulating the cell cycle pathway (Fig. 7). *Mdm2* were also up-regulated according to the p53 protein accumulation as negative feedback although *Mdm2* is negative regulator of the p53 pathway [21]. KEGG mapping also showed that reduced expression of *Cdk1*(*Cdc2a*) leads to down-regulation of *Plk1*, which has critical function during mitosis [24]. In addition, it could be found that *Espl1* having a role in chromosome segregation was inhibited by down-regulation of *Anapc5* and *Cdc20* in the cell cycle pathway (Fig. 7) [25]. *Mcm* genes, that were down-regulated after MFP treatment in this study, have been postulated to couple DNA replication to cell cycle progression [26, 27]. *Pola2*, *Pold1*, *Pold2*, *Pole*, *Pole3*, and *Prim2*, which are associated with DNA polymerases and participate in pathways of DNA replication, pyrimidine metabolism, and purine metabolism [28], were also down-regulated in this study. In addition, other genes related to pyrimidine and purine metabolisms were also down-regulated. Since pyrimidine and purine nucleotides are essential precursors for RNA and DNA synthesis [29], it could be postulated that DNA replication pathway were also attenuated by down-regulation of pyrimidine and purine metabolism. Subsequently, all above results showed that down-regulated genes by MFP treatment were mainly associated with pathway of cell cycle, DNA replication, and pyrimidine and purine metabolisms that are known to affect cell proliferation [21, 23, 26, 28, 29]. Based on the KEGG mapping, some of genes associated with cell cycle pathway including *Anapc5*, *Cdc20*, *Cdc25a*, *Cdc45l*, *Cdc6*, *Cdc7*, *Cdkn2c*, *Dbf4*, *E2f2*, *Espl1*, *Orc6l*, and *Plk1* were involved in cell growth as well as death. It could be considered that changes of expression level in these genes are more related to cell growth and/or inhibition of cell proliferation than apoptosis and/or necrosis, because RAW 264.7 cells treated with 100 μg/mL of MFP for 12 and 48 h showed high cell numbers in MTT assay compared with 0 h. Moreover, there was no significant difference among the cell numbers at 12, 24, and 48 h. In addition, the genes associated with cell cycle showing altered gene expression were mostly classified as cluster 2 showing pattern of continuous down-regulation. This result was supported by the MTT assay results (Fig. 2) that showed continuous low proliferation rate in MFP-treated RAW 264.7 cells compared to the nontreated cells. To sum up, it could suggest that RAW 264.7 cells showed decrease of cell proliferation, responding to MFP treatment.

**Fig 10:**
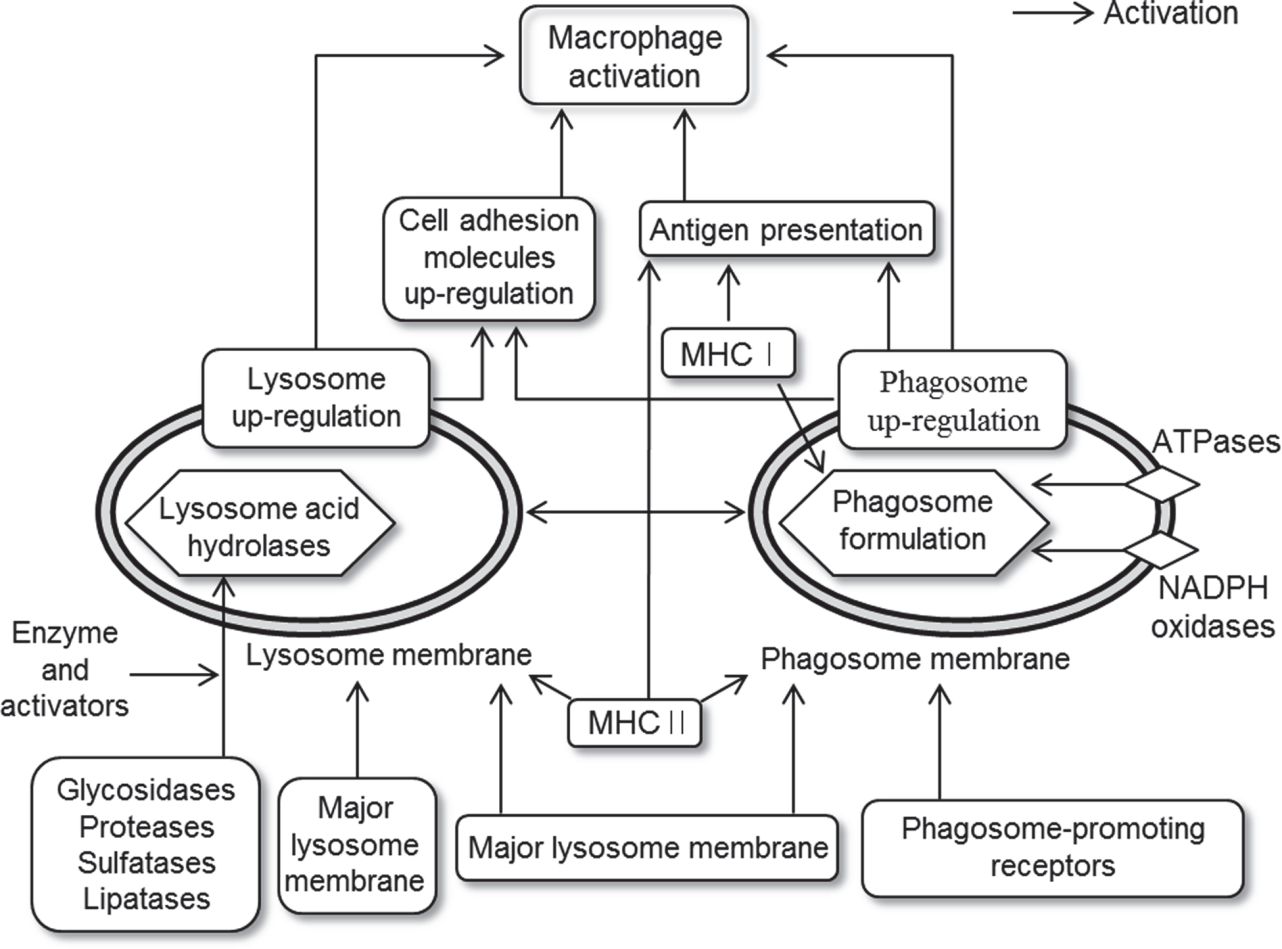
Suggesting diagram of activation pathways. This diagram was determined through the analysis of genes whose expression level was altered in RAW 264.7 cells that were treated with MFP 100 μg/mL. MFP treatment up-regulated the pathways of lysosome and phagosome. The pathway mapping was conducted using KEGG database.

KEGG mapping analysis revealed that up-regulated genes by MFP treatment were predominantly associated with the lysosome and phagosome pathways. A large number of genes involved the lysosome pathway were coupled with the phagosome pathway. Most of up-regulated genes in pathways of endocytosis and antigen processing and presentation were also involved in the phagosome pathway. Lysosomal acid hydrolases and lysosomal membrane proteins are essential for lysosome function [30, 31]. In this study, genes encoding lysosomal acid hydrolases and lysosomal membrane were up-regulated by MFP treatment. Lysosomal activities play an important role in the processing of peptides and degradation of biomacromolecules that bind MHC П molecules, and thereby enhance the activities of antigen receptors on T cells [31, 32]. This activities of antigen processing and presentation are crucial for immunity to pathogens [32]. Beside, lysosomes are crucial for the maturation of phagosome to phagolysomes in phagocytosis, which is important for cellular defense against pathogens [30]. As innate immunity, macrophage-driven phagocytosis initiates signaling cascades that connect the innate and adaptive immunity pathways to elicit a sustained immune response. Moreover, antigen presentation by macrophages is dependent on phagosome activity [33] and enhanced phagocytosis is an indicator of macrophage activation [10]. Corresponding with these results, KEGG mapping also showed that the phagosome pathway (Fig. 8) was up-regulated by MFP treatment. MFP treatment induced up-regulation of *Atp6v0a1*, *Atp6v1a*, *Atp6v1d*, and *Atp6v1g1*, which were known to play a critical role in receptor-mediated endocytosis by providing the acidic endosomal environment in phagosome [34]. We suggest that the up-regulation of lysosome- and phagosome-associated genes observed in this study may indicate enhanced macrophage activities, which in turn would enhance the responses of antigen presentation [32, 35, 36]. This is also supported by data showing the up-regulation of genes associated with cell adhesion molecules (*Pvrl2*, *Pvrl3*, *Cd40*, *Cd80*, *Cd86*, *Cd274*, *Itgb7*, and *Sdc3*) that are expressed on the cell surface and play a critical role in immune responses and inflammation [37]. Moreover, genes associated with antigen presentation such as *Cd74*, *Ctsl*, *Ctss*, *H2-D1*, *H2-K1*, *H2-Q6*, *H2-Q7*, *H2-Q8*, *H2-T23*, and *Hspa2* were also up-regulated by MFP treatment (Fig. 10). Previous studies showed that enhanced antigen presentation in macrophages can reinforce the activities of T and B cells, thereby strengthening the immune response [10, 38, 39]. In addition, the genes associated with the lysosome and phagosome pathways were mostly involved in cluster 1. However, genes associated with microtubule activity of the phagosome pathway showed different expression pattern classes. These latter classes included *Actb*, *Tfrc*, *Tuba1a*, *Tuba1b*, *Tubb2c*, *Tubb5*, and *Tubb6*, which were all down-regulated (Fig. 8). Microtubules are known to play roles in late phagosome maturation, such as fusion with endocytic organelles and phagolysosome formation [40]. Additionally, fusion of the phagosome with the lysosome and endosome in activated macrophages is delayed [33]. Therefore, down-regulation of microtubule expression may be indicative of early phagosome status in activated macrophages. In summary, the immune-enhancing effects of mica are likely induced, at least in part, by the activation of macrophages through up-regulation of the pathways mentioned above (Fig. 10).

These results could be believed as the specific responses of macrophages to MFP simulation. There were several studies on cell-particle interactions using a microarray tool [11, 41–43]. Water et al. (2009) demonstrated that RAW 264.7 cells showed altered regulation in genes expression associated with chemokines and inflammation, responding to stimulation of 500 nm of silica particles [11]. Murine cells isolated from branchoalveolar lavage containing macrophages, lymphocytes, and neutrophils showed different gene expression involved in immune, inflammatory, and cytoskeleton organization, responding to 580 nm of urban particulate matter [43]. In case of human macrophages, it was reported that altered gene expression related to cytokines and signal transduction was observed in U937 cells following the stimulation of polyethylene particles (1,710–2,580 nm) [42]. In addition, stimulation with fine ambient particles (2,500 nm) showed effect of gene modulation involved in metal binding and oxidative stress in human alveolar macrophages [41]. However, changes of cell systemic function including up-regulation of lysosome and phagosome pathways were not reported in previous that showed responses of cell to the fine particles stimulation. Therefore, it could be believed that the up-regulation of lysosome and phagosome pathway identified in this study result from the specific responses of macrophage to MFP stimulation, not common reaction against fine particles.

## Conclusions

In this study, the effects of MFP on gene expression profile were analyzed using RAW 264.7 murine macrophages, as this cell type plays a critical role in the immune system. Genes associate with pathways related to the cell cycle, DNA replication, and pyrimidine and purine metabolism, were down-regulated, consistent with a mica-dependent reduction of proliferation. Mica also up-regulated genes associated with lysosome and phagosome function (Fig. 10) in RAW 264.7 cells. We speculate that macrophage activation is due to up-regulation of some of these pathways, and by extension that the positive effects of mica in the immune system are mediated through increased lysosomal and phagosomal activity in macrophages.

## Supporting Information

S1 Table Clustering result of all gens showing altered expression after MFP treatment. (XLSX)

S2 Table Pathways determined using the differentially expressed genes classified as cluster 1 and 2. The pathway mapping was conducted using KEGG database.
(XLSX)
